# Developmental and geographic transcriptomic variation in *Anisakis simplex* (s. s.) reveals lncRNA-mediated regulation of mRNA expression

**DOI:** 10.1038/s41598-026-47984-8

**Published:** 2026-04-20

**Authors:** Robert Stryiński, Mateusz Maździarz, Mónica Carrera, Elżbieta Łopieńska-Biernat

**Affiliations:** 1 Department of Biochemistry, Faculty of Biology and Biotechnology, University of Warmia and Mazury in Olsztyn, Olsztyn, Poland; 2 Department of Botany and Nature Protection, Faculty of Biology and Biotechnology, University of Warmia and Mazury in Olsztyn, Olsztyn, Poland; 3 Department of Food Technology, Institute of Marine Research (IIM), Spanish National Research Council (CSIC), Vigo, Spain

**Keywords:** Anisakiasis, Transcriptome, mRNA, Long non-coding RNA, Populational differences, Computational biology and bioinformatics, Ecology, Ecology, Evolution, Genetics, Molecular biology

## Abstract

Marine nematodes of the genus Anisakis are shaped by complex life cycles and heterogeneous marine environments, yet the molecular basis of their developmental and population differentiation remains poorly understood. Here, we analyzed mRNA and long non-coding RNA expression in third- and fourth-stage larvae of *Anisakis simplex* (s. s.) collected from the Baltic Sea and the Northeast Atlantic using high-throughput RNA sequencing. Larval development was associated with extensive transcriptional remodeling involving cuticle organization, metabolism, proteolysis, and host-interaction pathways. Comparisons between populations revealed pronounced geographic divergence, including population-specific gene expression and reversal patterns shared across developmental stages. A subset of long non-coding RNAs was linked to the regulation of developmentally and population-biased genes, targeting pathways related to energy metabolism, ion transport, and protein synthesis. These findings demonstrate that developmental progression and geographic origin jointly shape the transcriptomic architecture of *Anisakis simplex* (s. s.), providing insight into molecular mechanisms underlying parasite adaptation with potential relevance for ecology, evolution, and anisakiasis risk.

**Supplementary Information:**

The online version contains supplementary material available at 10.1038/s41598-026-47984-8.

## Introduction

Diseases transmitted to humans through the consumption of seafood infected with bacteria, viruses, fungi, or parasites are collectively known as ichthyozoonoses. One of the most relevant and rapidly emerging ichthyozoonotic diseases affecting humans is anisakiasis, caused by marine nematodes of the genus Anisakis (Nematoda, Rhabditida, Ascaridomorpha: Anisakiadae). These nematodes parasitize the digestive tract of aquatic vertebrates, and their complex life cycle involves multiple hosts across different trophic levels^1,2^. The life cycle comprises four larval stages: L1–L2 in eggs, L3 in intermediate or paratenic hosts, and L4 (preadults) and adults in mammalian definitive hosts^3^. In general, marine mammals serve as final hosts, fish and squids act as intermediate or paratenic hosts, while planktonic crustaceans are the first intermediate hosts^4^.

The first reported human invasion was documented in the Netherlands ^5^. Humans acquire anisakiasis mainly by consuming raw or undercooked marine fish or cephalopods infected with larval stages of Anisakis species^6^. Three species of the genus are known to cause infections in humans: *A. simplex* sensu stricto (s. s.), *A. pegreffii*, and *A. physeteris*^4^. Increasing globalization, expanding international seafood markets, and the popularity of raw fish dishes in various countries, including Japan and those in Southern Europe, have contributed to rising exposure ^7–11^.

*Anisakis* invasion poses health risks because larvae can penetrate the gastrointestinal mucosa and induce inflammatory and allergic reactions^7,8^. Symptoms include abdominal pain, nausea, vomiting, and diarrhea and the Th2-type immune response is observed^9,12^. In severe cases, parasitic antigens can trigger anaphylactic shock^6,10^. Misdiagnosis is common due to symptom overlap with other gastrointestinal disorders^6,10,12^. Exposure to Anisakis larvae has also been reported as a potential risk factor for gastric or colon adenocarcinoma^13^.

Given these concerns, *A. simplex* is recognized as a biohazardous organism ^14–16^. Estimates suggested 76,000 cases of anisakidosis globally by 2017^16,17^, though this is likely underestimated, considering that Japan alone reports nearly 20,000 cases annually^18^. A meta-regression analysis revealed a 283-fold increase in the infection rate of fish and invertebrates by *Anisakis* spp. between 1962 and 2015^19^, underscoring the expanding distribution of these parasites and their growing relevance for human health and fisheries.

The distribution of marine helminths is shaped by hydrographic and climatic conditions^20^ and by trophic interactions among final, intermediate, and paratenic hosts. Globalization, climate change, and regulations aimed at protecting marine ecosystems are considered major drivers of increased transmission of Anisakis parasites^3,20–22^.

Currently, nine genetically recognized Anisakis species differ in geographic distribution and definitive host range^4,23,24^. *Anisakis simplex* exhibits a wide circumpolar distribution, inhabiting subarctic and temperate waters in both the Atlantic and Pacific Oceans^23^. Its range has expanded into formerly parasite-free regions, likely in response to climate change^3,20,21,25,26^. Additionally, the presence of hybrids between *A. simplex* (s. s.) and *A. pegreffii* has been documented^1,23,27^. These hybrids possess an expanded repertoire of allergen families^28^, suggesting that hybridization may facilitate adaptation to changing environmental conditions.

Changes in the biology and epidemiology of Anisakis species may be linked to environmental shifts, potentially promoting geographical expansion and increased host invasion rates. Such processes are likely influenced by molecular changes in the transcriptome and proteome of the parasites. Local adaptation, host-driven selection, evolutionary divergence, and speciation processes depend on gene flow and genetic drift across parasite populations. Therefore, examining molecular differences between populations from distinct geographic regions, and identifying genes differentially expressed according to zoogeographic distribution, is essential for understanding microevolutionary dynamics in these organisms^29^.

With this background, the present study aimed to investigate the transcriptomic differences associated with larval development and geographic origin in *A. simplex* (s. s.). Specifically, we compared gene expression profiles between the third (L3) and fourth (L4) larval stages collected from two environmentally distinct regions: the Galician coast of the Northeast Atlantic Ocean (Ría de Vigo, Spain) and the Polish coast of the southeastern Baltic Sea (Władysławowo, Poland). Using high-throughput RNA sequencing, we analyzed both protein-coding transcripts (mRNAs) and long non-coding RNAs (lncRNAs) to identify differentially expressed mRNAs (DEGs), differentially expressed lncRNAs (DELs), and potential regulatory relationships between coding and non-coding transcripts. By integrating developmental and population-level comparisons, this study aims to provide new insights into the molecular mechanisms underlying larval development, geographic variation, and potential regulatory processes in *A. simplex* (s. s.), contributing to a better understanding of the ecological and evolutionary factors shaping parasite populations.

## Results

### Statistics of RNA sequencing

The sequencing generated 846,678,718 raw paired-end reads on average 65.12 million per sample with Q20 value on average 99.99%. The short reads, low-quality reads and ambiguous sequences were removed from the raw reads, leaving on average 54,290,037 valid reads per sample, that were used for further analyses. The filtered reads were mapped to the PRJEB496 version of the *A. simplex* genome with mapped average rate 42.25% (Tables 1, 2).

**Table 1:** Raw data obtained after quality control.

Sample	Raw data		Valid data		Valid ratio reads (%)	Q20 (%)	GC content (%)
	Read	Base	Read	Base			
BAL L3	63,544,162	9.77 G	58,046,832	8.70 G	83,61	99,99	53,63
BAL L4	65,173,631	9.53 G	53,094,270	7.96 G	89,08	99,99	53
ATL L3	66,616,371	9.99 G	51,755,605	7.76 G	77,70	99,99	55
ATL L4	65,342,068	9.80 G	54,263,444	8.14 G	83,05	99,99	51,5

**Table 2:** Statistics of mapping to the reference genome.

Sample	Valid reads	Mapped reads (%)	Unique mapped reads (%)	Multi mapped reads (%)	PE mapped reads (%)	Reads map to sense strand (%)	Reads map to antisense strand (%)	Non-splice reads (%)	Splice reads (%)
BAL L3	58,046,832	36.90	23.69	13.20	24.72	15.38	16.22	22.80	8.81
BAL L4	53,094,270	45.60	29.29	16.31	31.41	18.74	19.50	27.20	12.28
ATL L3	51,755,605	41.94	26.42	15.52	26.51	18.15	20.33	25.35	10.35
ATL L4	54,263,444	44.54	32.32	12.22	26.66	19.61	20.41	28.71	11.31

### Identification of DEGs and contrasting expressed DEGs

**Fig. 1:**
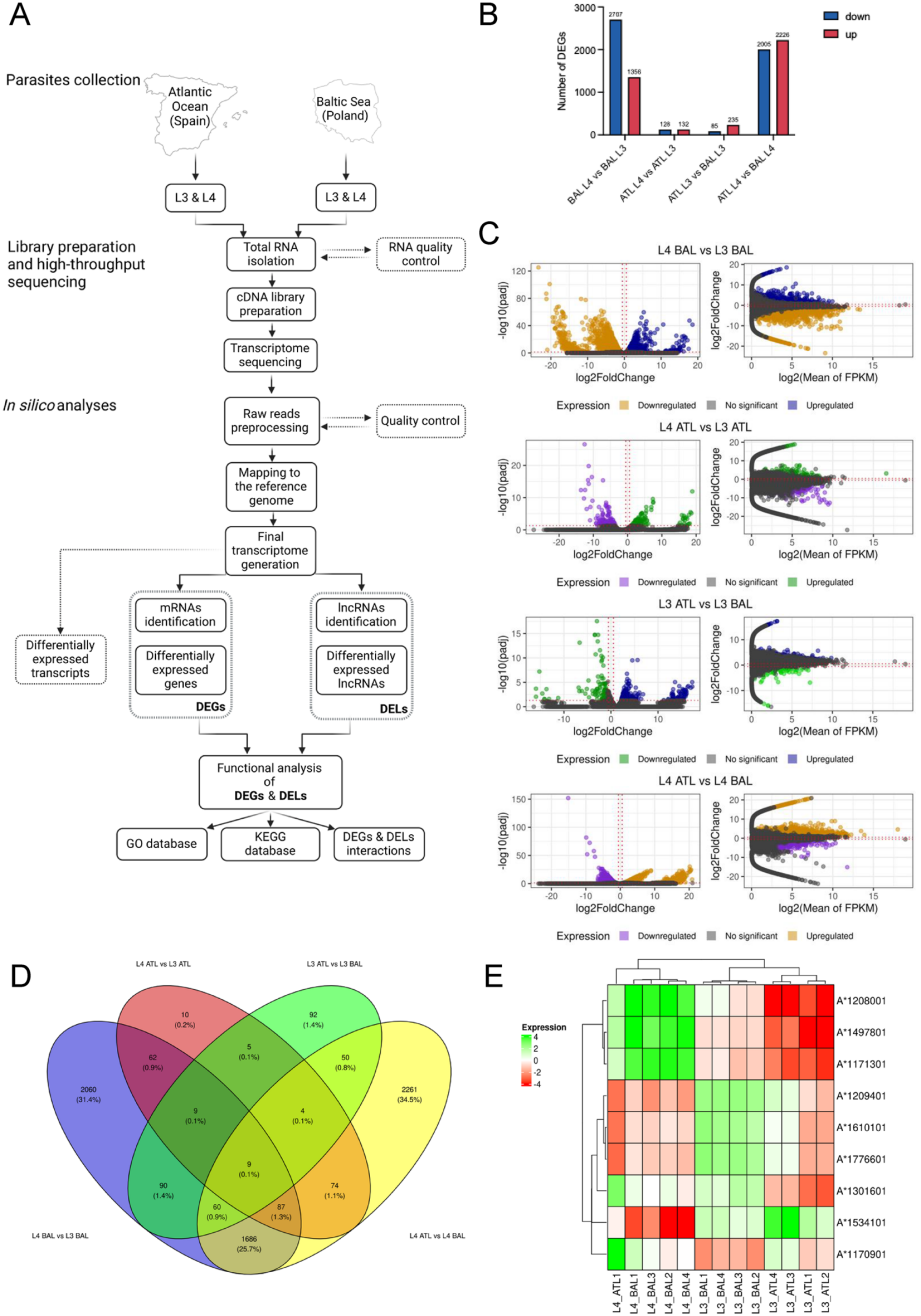
Comparative transcriptomic analysis of *A. simplex* s. s. larvae reveals stage- and region-specific RNA expression patterns. (**A**) Schematic overview of the experimental design and bioinformatic workflow used for transcriptomic comparison of *A. simplex* s. s. third-stage (L3) and fourth-stage (L4) larvae collected from two geographical regions: the Baltic Sea (Poland) and the Atlantic Ocean (Spain). Larvae were subjected to total RNA isolation, RNA quality assessment, cDNA library preparation, and high-throughput RNA sequencing. After quality filtering and mapping of raw reads to the reference genome, final transcriptomes were generated. Differentially expressed genes (DEGs) and long non-coding RNAs (DELs) were identified and functionally annotated using Gene Ontology (GO) and KEGG pathway databases. Created with BioRender.com (**B**) Numbers of significantly upregulated and downregulated DEGs identified in pairwise comparisons between developmental stages (L3 vs. L4) and geographical regions (Baltic vs Atlantic). Differential expression was determined using a threshold of |log_2_ FC|≥ 1 and an adjusted *p* value < 0.05. (**C**) Volcano plots (left) and MA plots (right) illustrating differential gene expression for pairwise comparisons: L4 BAL vs L3 BAL, L4 ATL vs L3 ATL, L3 ATL vs L3 BAL, and L4 ATL vs L4 BAL. Genes with significant upregulation or downregulation (|log_2_ FC|≥ 1, adjusted *p* value < 0.05) are highlighted, while non-significant genes are shown gray. (**D**) Venn diagram showing the overlap and uniqueness of DEGs among the four pairwise comparisons, highlighting shared transcriptional responses as well as genes specifically associated with larval development stage or geographical origin. (**E**) Hierarchical clustering heatmap of selected DEGs across all experimental groups (L3 and L4 larvae from the Baltic Sea and Atlantic Ocean). Expression levels are shown as normalized values, illustrating distinct clustering patterns driven by developmental stage and sampling region.

Differential expression analysis revealed extensive transcriptional differences between developmental stages and between the two geographical populations (Fig. 1A). In L4 BAL vs. L3 BAL, a total of 4063 DEGs were detected (1356 upregulated and 2707 downregulated). The L4 ATL vs. L3 ATL comparison contained 260 DEGs (132 upregulated and 128 downregulated). At equivalent developmental stages, L3 ATL vs. L3 BAL yielded 319 DEGs (234 upregulated and 85 downregulated), while L4 ATL vs. L4 BAL produced 4231 DEGs (2226 upregulated and 2005 downregulated) (Fig. 1B). Expression patterns for all comparisons are presented in volcano and MA plots (Fig. 1C, Supplementary File 1).

A set of nine DEGs was shared across all four comparisons (Fig. 1D). These included genes encoding a *PTC-related protein* (ASIM_0001170901), *phosphoenolpyruvate carboxykinase (GTP)* (ASIM_0001171301), an *M20_dimer domain-containing protein* (ASIM_0001208001), *secreted protein* (ASIM_0001209401), *ubiquitin-like domain-containing protein* (ASIM_0001301601), cuticle-associated *Col_cuticle_N protein* (ASIM_0001497801), *SWEET sugar transporter* (ASIM_0001534101), *VWFA domain-containing protein* (ASIM_0001610101), and an *SSD domain-containing protein* (ASIM_0001776601) (Supplementary File 1).

Population-specific transcriptional signatures were observed in both developmental stages (Fig. 1D). In the L3 ATL vs. L3 BAL comparison, 92 DEGs were unique to this comparison. Among these, *DUF3402 domain* (ASIM_0001882401; log_2_FC =  + 17.25) and *DNA_pol3_finger domain* (ASIM_0000550701; log_2_FC =  + 16.96) showed the highest expression in L3 ATL, whereas *EGF-like domain* (ASIM_0000563901; log_2_FC =  − 15.93) and *ovule protein* (ASIM_0000936701; log_2_FC =  − 15.93) were more abundant in L3 BAL.

In the L4 ATL vs. L4 BAL comparison, 2,261 DEGs were unique. The transcripts with the highest relative expression in L4 ATL included *cystatin domain* (ASIM_0001203001; log_2_FC =  + 19.50) and *transmembrane protein* (ASIM_0000707401; log_2_FC =  + 19.27). Conversely, *choline O-acetyltransferase* (ASIM_0001650901; log_2_FC =  − 6.23) and *glucosamine-6-phosphate deaminase* (ASIM_0002043001; log_2_FC =  − 5.93) were more highly expressed in L4 BAL. A complete list of DEGs, including fold-changes, FPKM expression values, and gene annotations, is provided in Supplementary File 1.

A defined set of nine DEGs shared across all four comparisons (Fig. 1D), that is, common to both larval stages and to the Baltic and Atlantic populations of *A. simplex* s. s., showed consistent patterns of reversal expression. These genes displayed opposite regulatory directions depending on developmental stage and geographical origin (Fig. 1E).

Within individual developmental stages, several of the shared DEGs demonstrated contrasting expression profiles between stages and/or populations. In the Baltic group, *M20 dimer domain-containing protein* (ASIM_0001208001), *Col_cuticle_N domain-containing protein* (ASIM_0001497801), and *phosphoenolpyruvate carboxykinase (GTP)* (ASIM_0001171301) were more expressed in the L4 and less expressed in L3 stage, whereas in Atlantic group the same genes showed the similar trend. Other shared DEGs, including *secreted protein* (ASIM_0001209401), *VWFA domain-containing protein* (ASIM_0001610101), *SSD domain-containing protein* (ASIM_0001776601), *ubiquitin-like domain-containing protein* (ASIM_0001301601) were upregulated in Baltic L3 larvae (BAL L3) and their expression remained low or downregulated in Atlantic L3 larvae (ATL L3), with the strongest contrast observed in expression of a *ubiquitin-like domain-containing protein* (ASIM_0001301601). The expression of a *SWEET sugar transporter* (ASIM_0001534101) was lower in BAL L4, than in ATL L4. The similar pattern has been shown for *PTC-related protein* (ASIM_0001170901), where the expression was higher for L4 larvae from Atlantic, than in BAL L4 (Fig. 1E). These examples of contrasting expression patterns were present in each of the four DEG comparisons, indicating that the same transcripts change direction of regulation depending on both larval stage (L3 vs. L4) and/or population (Baltic vs. Atlantic). Although some variability between biological replicates was observed, differential expression analysis confirmed statistically significant and consistent differences at the group level. The full list of nine shared DEGs together with their expression values across all samples is provided in Supplementary File 1.

To validate the RNA-seq results, selected genes were analyzed using RT-qPCR (Supplementary Fig. 1). Overall, the RT-qPCR results reproduced the general direction of expression changes observed in the RNA-seq dataset. Although the magnitude of fold changes differed for some genes, such discrepancies are commonly observed due to methodological differences between sequencing-based quantification and targeted PCR-based approaches. These results support the reliability of the RNA-seq–derived expression patterns.

### Functional enrichment analysis of DEGs

**Fig. 2:**
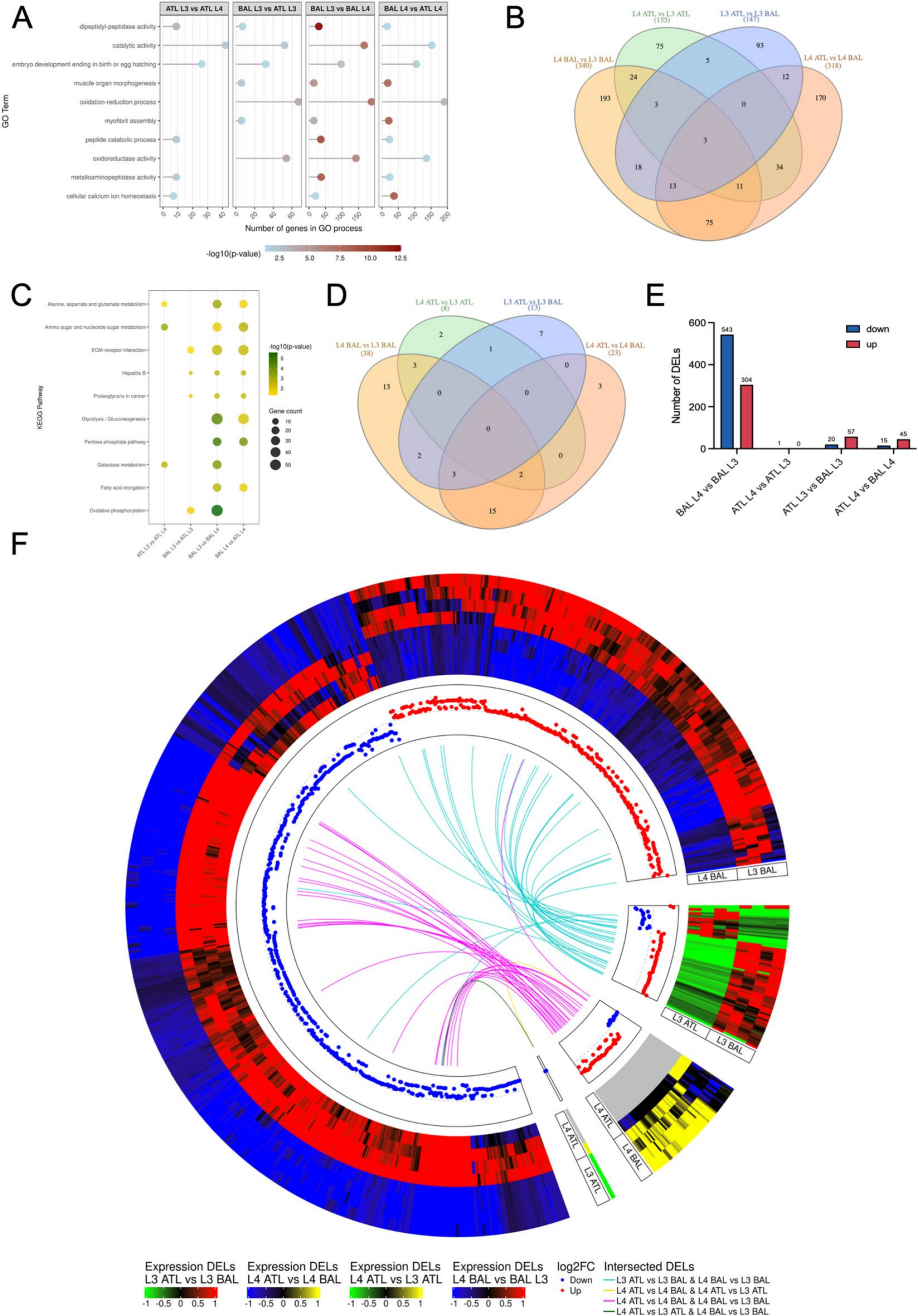
Functional enrichment of DEGs and expression patterns of DELs in *A. simplex* s. s. larvae. (**A**) Dot plot summarizing Gene Ontology (GO) enrichment analysis of DEGs identified in four pairwise comparisons: L4 BAL vs L3 BAL, L4 ATL vs L3 ATL, L3 ATL vs L3 BAL, and L4 ATL vs L4 BAL. The x-axis represents the number of DEGs assigned to each GO term, while color intensity indicates statistical significance expressed as -log₁₀(p value). Enrichment includes terms from the biological process (BP), molecular function (MF), and cellular component (CC) categories. Details can be found in Supplementary File 2. (**B**) Venn diagram illustrating the overlap of significantly enriched GO terms among the four DEG comparisons. Only three GO terms: catalytic activity (GO:0003824), dipeptidyl-peptidase activity (GO:0008239), and embryo development ending in birth or egg hatching (GO:0009792), were shared across all comparisons, whereas the majority of significant GO terms were comparison-specific. (**C**) KEGG pathway enrichment analysis of DEGs across the four pairwise comparisons. Dot size corresponds to the number of DEGs mapped to each pathway, and color intensity represents pathway significance (–log₁₀(p value)). Selected enriched pathways are shown for each comparison. Details can be found in Supplementary File 3. (**D**) Venn diagram showing the distribution of significantly enriched KEGG pathways across comparisons. No KEGG pathway was shared among all four datasets, indicating distinct pathway-level responses associated with larval developmental stage and geographical origin. (**E**) Numbers of significantly upregulated and downregulated DELs identified in each comparison. Differential expression was defined as |log₂ FC|≥ 1 with an adjusted p value < 0.05. (**F**) Circos plot integrating expression profiles and shared DELs across all comparisons. Outer heatmap tracks represent normalized DEL expression levels, with red and blue indicating upregulation and downregulation, respectively. Inner tracks show log₂ FC values, and connecting links indicate DELs shared between comparisons related to larval stage and geographical population.

Gene Ontology (GO) enrichment analysis was performed for all four pairwise comparisons. The total number of annotated GO terms and the number of significant GO terms differed among datasets (Fig. 2A, Supplementary File 2). For L4 BAL vs. L3 BAL, 4055 GO terms were identified, of which 340 were significant (BP: 157; MF: 133; CC: 50). In L4 ATL vs. L3 ATL, 1984 GO terms were detected, including 155 significant terms (BP: 75; MF: 48; CC: 32). The population-level comparison L3 ATL vs. L3 BAL included 2,224 GO terms, with 147 classified as significant (BP: 68; MF: 53; CC: 26). For L4 ATL vs. L4 BAL, a total of 4,875 GO terms were annotated, including 318 significant categories (BP: 158; MF: 94; CC: 66).

Across all comparisons, a small subset of GO terms was shared. These included three terms shared across all four comparisons: catalytic activity (GO:0003824; MF), dipeptidyl-peptidase activity (GO:0008239; MF), and embryo development ending in birth or egg hatching (GO:0009792; BP) (Fig. 2A, Supplementary File 2). Many GO terms were unique at the level of significance to individual comparisons (Fig. 2B, Supplementary File 2). Examples include release of sequestered calcium ion into cytosol (GO:0051209; BP), observed only in L4 ATL vs. L4 BAL; structural constituent of cuticle (GO:0042302; MF), identified exclusively in L4 ATL vs. L3 ATL; and response to stress (GO: 0006950; BP), found significant solely in L4 BAL vs. L3 BAL (Supplementary File 2). The distribution of representative significant GO terms for each comparison is illustrated in Fig. 2B.

To investigate pathway-level differences, KEGG enrichment analysis was performed for all four comparisons. The number of significantly enriched KEGG pathways, based on unique pathway identifiers, differed across datasets. In L4 BAL vs. L3 BAL, 38 pathways were significantly enriched. The L4 ATL vs. L3 ATL comparison included 8 significant pathways, while L3 ATL vs. L3 BAL contained 13 significant pathways. For L4 ATL vs. L4 BAL, 23 KEGG pathways met the significance threshold. The visualization in Fig. 2C summarizes selected enriched pathways across the four comparisons (Supplementary File 3).

Several KEGG pathways were found to be exclusive to individual comparisons (Fig. 2D, Supplementary File 3). Representative examples included ribosome (ko03010), histidine metabolism (ko00340), and pentose and glucuronate interconversions (ko00040) in L4 BAL vs. L3 BAL; glycosylphosphatidylinositol(GPI)-anchor biosynthesis (ko00563), and basal transcription factors (ko03022) in L4 ATL vs. L3 ATL; glutamatergic synapse (ko04724), fatty acid biosynthesis (ko00061), retrograde endocannabinoid signaling (ko04723), and lysine degradation (ko00310) in L3 ATL vs. L3 BAL; and mismatch repair (ko03430), MAPK signaling pathway (ko04010), and other glycan degradation (ko00511) in L4 ATL vs. L4 BAL. No KEGG pathway was shared across all four comparisons.

### Differentially expressed long non-coding RNAs

**Fig. 3:**
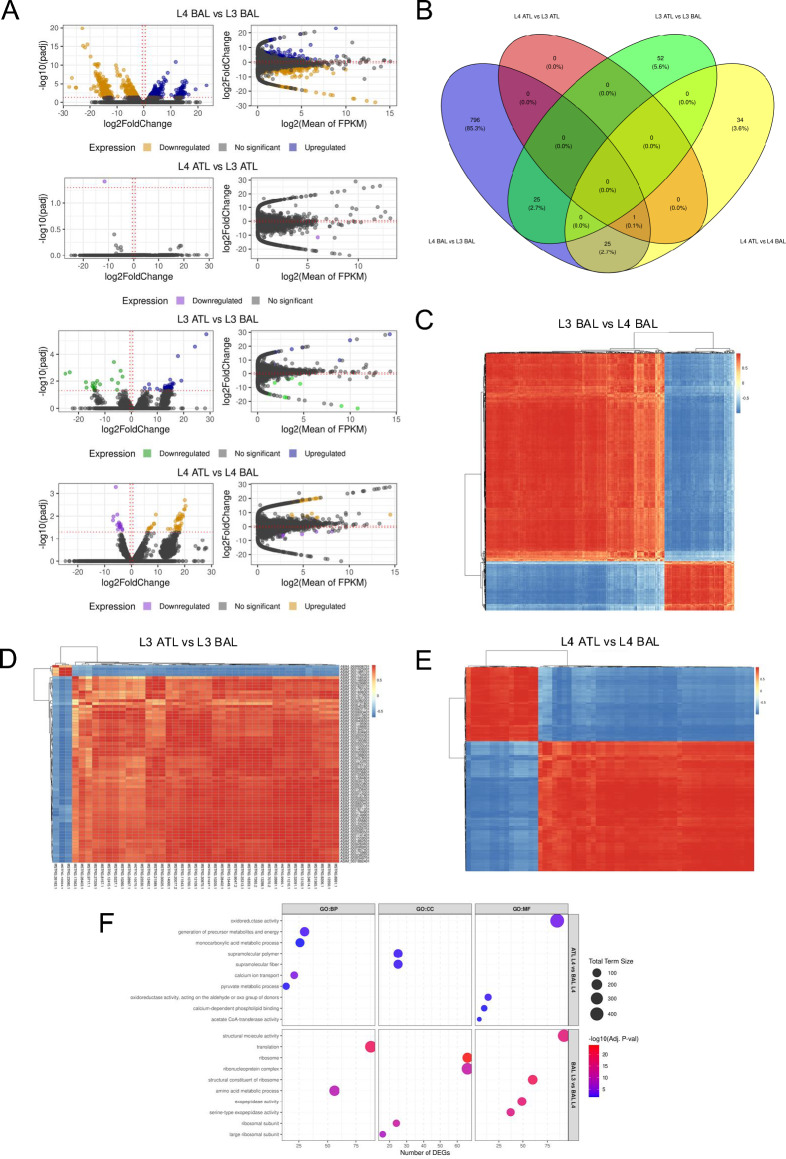
Differential expression, overlap, and regulatory interactions of DELs in *A. simplex* s. s. larvae. (**A**) Volcano plots (left) and MA plots (right) illustrating differential expression of DELs across four pairwise comparisons: L4 BAL vs L3 BAL, L4 ATL vs L3 ATL, L3 ATL vs L3 BAL, and L4 ATL vs L4 BAL. Significantly upregulated and downregulated DELs were identified using a threshold of |log_2_ FC|≥ 1 and an adjusted *p* value < 0.05, while non-significant transcripts are shown gray. (**B**) Venn diagram showing the overlap of DELs among the four comparisons. No DELs were shared across all datasets, indicating strong comparison-specific expression patterns. The developmental comparison within the Baltic population (L4 BAL vs L3 BAL) contained the largest number of unique DELs. Details can be found in Supplementary File 4. (**C**–**E**) Correlation matrices illustrating regulatory interactions between DELs and their predicted target differentially expressed genes (DEGs) across three comparisons: L4 BAL vs L3 BAL (**C**), L3 ATL vs L3 BAL (**D**), and L4 ATL vs L4 BAL (**E**). Color intensity reflects correlation strength. The Baltic developmental comparison exhibits a dense and highly interconnected regulatory network, whereas population-level comparisons display fewer, more specific DEL–DEG interactions. (**F**) Gene Ontology (GO) enrichment analysis of DEGs predicted to be regulated by DELs. Enriched GO terms are shown for biological process (BP), cellular component (CC), and molecular function (MF) categories. Dot size reflects the total number of genes associated with each term, while color intensity indicates statistical significance expressed as –log_10_ (adjusted p value). GO enrichment was observed primarily in the L4 BAL vs L3 BAL and L4 ATL vs L4 BAL comparisons, whereas no significant GO terms were detected for the remaining datasets. Details can be found in Supplementary File 4.

The analysis of DELs revealed regulatory and expression divergence between developmental stages and geographical populations (Fig. 2E, Supplementary File 4). In L4 BAL vs. L3 BAL, a total of 847 DELs were detected, including 304 upregulated and 543 downregulated transcripts. Only one DEL was identified in L4 ATL vs. L3 ATL, which was downregulated. The population-level comparisons showed moderate DEL variation: L3 ATL vs. L3 BAL contained 77 DELs (57 upregulated; 20 downregulated), while L4 ATL vs. L4 BAL comprised 60 DELs (45 upregulated; 15 downregulated). The overall distribution and magnitude of differential expression are illustrated by the volcano and MA plots (Fig. 3A), which display the relationship between fold change, statistical significance, and expression levels for each comparison. These visualizations emphasize the pronounced transcriptional shift observed in the L4 BAL vs. L3 BAL comparison, where the largest number of DELs was detected, whereas the L4 ATL vs. L3 ATL comparison showed minimal expression divergence. The circos plot (Fig. 2F) provides an integrated overview of DEL expression patterns and their predicted regulatory links with DEGs, highlighting the markedly denser regulatory network detected in the Baltic developmental comparison relative to the other contrasts.

The overlap among DELs across comparisons was minimal (Fig. 3B). No DELs were shared among all four comparisons, indicating a high degree of comparison-specific regulatory activity. Developmental DEL variation in the Baltic population was particularly extensive, with 796 DELs (85.3%) unique to L4 BAL vs. L3 BAL. In contrast, only 52 DELs were unique to L3 ATL vs. L3 BAL, and 34 DELs uniquely characterized L4 ATL vs. L4 BAL. Only a single DEL overlapped between the Atlantic and Baltic comparisons (Fig. 3B, Supplementary File 4).

The potential regulatory relationships between DELs and DEGs were quantified across all four comparisons. In L4 BAL vs. L3 BAL, 847 DELs were identified in total, of which 630 DELs were linked to 1696 unique DEGs, forming 12,312 DEL–DEG predicted associations (Fig. 3C). In L3 ATL vs. L3 BAL, 77 DELs were detected, but only 43 DELs showed potential regulatory connections, collectively targeting 69 DEGs and producing 116 DEL–DEG pairs (Fig. 3D). Similarly, in L4 ATL vs. L4 BAL, 60 DELs were present, with all of them engaging in potential regulatory interactions with 1787 DEGs, resulting in 8369 pairs (Fig. 3E). In contrast, the developmental comparison L4 ATL vs. L3 ATL contained a single DEL (MSTRG.540.1), which showed 18 potential interactions with 18 different DEGs, indicating the lowest regulatory activity in this group.

A comprehensive visualization of all DEL–DEG potential relationships is provided in the correlation matrices (Fig. 3C–E), which highlights the dense regulatory structure of the Baltic developmental comparison (Fig. 3C) and the limited, highly specific, correlated expression patterns observed in the remaining population-related comparisons. All DELs and their predicted associated DEGs, including correlation values are provided in Supplementary File 5.

### Functional annotation of DELs-regulated DEGs

Gene Ontology (GO) enrichment analysis was performed exclusively for DEGs that were predicted to be regulated by DELs (Fig. 3F, Supplementary File 6). In the population-level comparison L4 ATL vs. L4 BAL, a total of 18 GO terms were significantly enriched, including 10 biological processes (BP), 4 cellular component (CC), and 4 molecular function (MF) terms. Enriched BP terms included calcium ion transport (GO:0006816), generation of precursor metabolites and energy (GO:0006091), and pyruvate metabolic process (GO:0006090). Cellular component enrichment was dominated by structural assemblies such as supramolecular polymer (GO:0099081), supramolecular fiber (GO:0099512), and supramolecular complex (GO:0099080). Molecular function terms associated with DEL-regulated DEGs included oxidoreductase activity (GO:0016491), oxidoreductase activity acting on aldehyde or oxo groups of donors (GO:0016903), and calcium-dependent phospholipid binding (GO:0005544).

In the developmental comparison L4 BAL vs. L3 BAL, GO enrichment revealed a substantially broader functional landscape, with 75 significantly enriched GO terms, comprising 39 BP, 11 CC, and 25 MF categories. Among BP terms, processes related to protein biosynthesis and metabolism were predominant, including translation (GO:0006412), amino acid metabolic process (GO:0006520), and oxoacid metabolic process (GO:0043436). Cellular components terms were largely associated with translational machinery, such as ribosome (GO:0005840), ribosomal subunit (GO:0044391), and ribonucleoprotein complex (GO:1990904). Consistently, enriched MF terms included structural constituent of ribosome (GO:0003735), structural molecule activity (GO:0005198), and exopeptidase activity (GO:0008238). Analysis did not show any significant GO terms for another two comparisons. Similarly, KEGG enrichment analysis did not reveal significant pathways for any of the comparisons examined.

## Discussion

In recent decades, marine nematodes have been increasingly exposed to a wide range of biotic and abiotic factors that profoundly influence their biology, distribution, and host–parasite interactions. Changes in water temperature, salinity, oxygen availability, trophic structure, and host composition driven by climate change, anthropogenic pressure, and ecosystem reorganization have been shown to affect parasite transmission dynamics and population structure^21,30–32^. These environmental pressures shape parasite ecology at the organismal level and may also induce molecular responses, including shifts in gene expression and its regulation, activation of metabolic pathways, and in general transcriptome composition. In parasites with complex life cycles, such as *A. simplex* s. s., these molecular adjustments are expected to be particularly pronounced during key developmental transitions (L3 and L4) and across geographically distinct populations experiencing different environmental regimes. However, the extent to which developmental stage and geographic origin jointly shape transcriptomic architecture in *A. simplex* s. s., including both, mRNA and lncRNA, remains poorly understood.

The Baltic Sea and the Northeast Atlantic represent markedly different marine environments in terms of temperature and salinity, both of which are key abiotic factors influencing parasite development and physiology^23,33^. Coastal waters of the southern Baltic Sea, including the Polish coast near Władysławowo, are characterized by relatively low mean annual sea-surface temperatures of approximately 9–11 °C, with strong seasonal variability and prolonged periods of cold conditions^34,35^. In contrast, the Ría de Vigo (NE Atlantic) experiences substantially warmer conditions, with mean annual sea-surface temperatures of approximately 16–18 °C, reflecting the influence of temperate Atlantic waters and regional upwelling dynamics^36,37^. This results in an average temperature difference of roughly 6–8 °C between the two sampling regions. Salinity differences between the two environments are even more pronounced. The Baltic Sea is a brackish water body with surface salinities typically ranging from 6 to 8 PSU (practical salinity unit) in the southern basins, due to limited water exchange with the North Sea and high freshwater input^38,39^. By contrast, the outer Ría de Vigo is influenced by open Atlantic waters and exhibits near-oceanic salinities of approximately 34–36 PSU, although lower values may occur locally in inner ría areas due to riverine input^37^. Consequently, the Atlantic sampling site has salinities approximately 27–30 PSU higher than those observed in the Baltic Sea. These strong and persistent environmental gradients provide a plausible ecological framework for the population-specific and stage-dependent transcriptomic differences observed in *A. simplex* s. s., including differential regulation of metabolic pathways and stress response mechanisms.

The present study offers a comprehensive transcriptomic comparison of *A. simplex* s. s. developmental stages and geographically distinct populations, integrating both coding and non-coding RNA expression profiles. Previous transcriptomic studies have demonstrated pronounced tissue-specific or stage-specific transcriptional differences between L3 and L4 larvae of *A. simplex*. Cavallero et al.^40^ used high-throughput RNA-Seq to characterize the transcriptomes of invasive larvae of *A. simplex* (s. s.) and *A. pegreffii*, focusing specifically on pharyngeal tissues as key anatomical sites involved in tissue penetration and host interaction. By combining whole-larva and tissue-specific transcriptomic profiling, the authors distinguished transcripts broadly expressed throughout the larval body from those selectively enriched in the pharyngeal region. Differential expression analyses identified hundreds of transcripts upregulated in the pharyngeal tissues of both species, many encoding proteolytic enzymes, anticoagulants, anesthetic-like molecules, inhibitors of host hemostasis, and immunomodulatory factors. These findings provided the molecular inventory of Anisakis transcripts potentially involved in tissue invasion and early host–parasite interactions. Study of Kim et al.^41^ showed the transcriptomic profile of L3 and L4 stage larvae and pointed out that polyubiquitin’s-related genes, collagen-related genes and mitochondrial enzyme-related genes were highly expressed both in L3 and L4. Among the DEGs, 675 were up-regulated in L3, while 1015 were up-regulated in L4. Authors additionally showed that several protease-related and protein biosynthesis-related genes were highly expressed in L3, all of which are thought to be crucial for invading host tissues. Collagen synthesis-related genes were highly expressed in L4, reflecting active biosynthesis of collagens during molting process^41^.

Our findings substantially extend these observations by including geographic comparisons and regulatory RNA profiling. In both Baltic and Atlantic populations, developmental stage comparisons identified hundreds or thousands of DEGs, enriched in biological processes such as cuticle synthesis and remodeling, energy metabolism, proteolysis, and signaling pathways related to host interaction. These functional categories correspond closely with the morphological and physiological transitions between L3 and L4 larvae, including preparation for host invasion, molting, adaptation to distinct host internal environments, and host–parasite interactions ^42–46^. Similar developmental restructuring has been reported in other parasitic nematodes, where transcriptional changes reflect preparation for new ecological niches^47^.

Geographic comparisons revealed equally striking transcriptomic divergence. Baltic and Atlantic larvae displayed distinct population-specific expression profiles, including differences in number of DEGs and numerous genes with contrasting expression profiles, upregulated in one population but downregulated in the other. Importantly, these reversals did not reflect a uniform Baltic–Atlantic contrast but rather gene-specific expression shifts that appeared differently across developmental stages and populations. It should also be noted that host species and tissue environment may influence parasite gene expression. In the present study, larvae were obtained from different fish hosts (Atlantic hake and Baltic herring), which differ substantially in ecology, physiology, and habitat conditions. Such host-related differences may affect nutrient availability, immune exposure, and microenvironmental factors experienced by the larvae, potentially contributing to some of the transcriptional variation observed between populations. Although our analysis primarily focused on developmental stage and geographic origin, host-associated effects should therefore be considered as an additional ecological factor shaping gene expression patterns in *A. simplex* (s. s.), as host-dependent transcriptional plasticity has been reported for a variety of parasites ^48–52^. To further assess the biological relevance of the experimental system, we compared transcriptomic profiles of in vitro–derived L4 larvae from Atlantic population with those obtained from L4 larvae collected directly from a natural host, striped dolphin (*Stenella coeruleoalba*). This analysis revealed a strong positive correlation in gene expression patterns (Pearson R = 0.7959), indicating that the in vitro model captures a substantial proportion of the transcriptional landscape observed in naturally developed larvae (Supplementary Fig. 2). These findings support the suitability of the in vitro culture system for investigating developmental transcriptomic changes in *A. simplex* (s. s.), while also reinforcing the interpretation of population-level differences observed in the present study.

Within the Baltic population, several DEGs, including the *M20 dimer domain-containing protein* (ASIM_0001208001), the *Col_cuticle_N domain-containing protein* (ASIM_0001497801), and *phosphoenolpyruvate carboxykinase (PEPCK)* (ASIM_0001171301) were more highly expressed in L4 larvae and showed lower expression in L3 larvae. A similar stage-dependent trend was observed in the Atlantic population, indicating that for these genes the primary axis of regulation is associated with larval development rather than population origin. These transcripts are linked to metabolic (PEPCK is a key enzyme in gluconeogenesis) and structural functions, suggesting conserved developmental remodeling processes during the L3 to L4 transition in *A. simplex* s. s.

In contrast, other shared DEGs exhibited population-dependent expression differences within the same developmental stage. Several transcripts, including a *secreted protein* (ASIM_0001209401), a *VWFA domain-containing protein* (ASIM_0001610101), an *SSD domain-containing protein* (ASIM_0001776601), and a *ubiquitin-like domain-containing protein* (ASIM_0001301601), were upregulated in Baltic L3 larvae, while their expression remained low or was downregulated in Atlantic L3 larvae. Among these, the *ubiquitin-like domain-containing protein* (ASIM_0001301601) showed the greatest contrast between populations, highlighting pronounced transcriptional divergence at the L3 ATL vs L3 BAL. Functionally, these proteins are generally associated with key cellular processes including ubiquitin-mediated protein turnover, lipid or sterol sensing, and extracellular matrix or host-interaction mechanisms mediated by von Willebrand factor A domains. Together, these functional classes suggest that population-level transcriptional differences at the L3 stage may involve modulation of protein regulation pathways, metabolic sensing, and parasite–host interface processes ^53–55^.

Population-specific expression differences observed at the L4 stage for selected shared DEGs may reflect distinct physiological strategies shaped by contrasting environmental conditions in the Baltic Sea and the Northeast Atlantic. The lower expression of the *SWEET sugar transporter* (ASIM_0001534101) in Baltic L4 larvae compared with Atlantic L4 larvae suggests potential differences in carbohydrate uptake or intracellular sugar allocation between populations. SWEET transporters mediate facilitated diffusion of mono- and disaccharides across cellular membranes and have been implicated in energy homeostasis and metabolic flexibility across a wide range of organisms, including *A. simplex*, where *sweet-1* expression was confirmed in both L3 and L4 stages^56^. Reduced expression of sugar transport–related transcripts in Baltic larvae may reflect altered metabolic demands under colder and less saline conditions, as temperature-dependent metabolic modulation has been widely documented in nematodes and other ectothermic parasites ^57–60^.

Conversely, the higher expression of the *PTC-related protein* transcript (ASIM_0001170901) in Atlantic L4 larvae may be associated with increased signaling activity during late larval development. Proteins related to the Patched (PTC) family are core components of the Hedgehog signaling pathway, which plays a central role in developmental patterning, tissue differentiation, and growth regulation in metazoans, including nematodes. Increased expression of *PTC-related protein* transcripts in Atlantic larvae may indicate intensified developmental signaling, potentially facilitating faster growth, tissue remodeling, or adaptation to warmer and more stable oceanic conditions^61,62^. Such enhanced signaling activity at the L4 stage could support accelerated developmental progression or improved physiological readiness for molting and stage transition. Taken together, these contrasting expression patterns suggest that while core developmental programs are conserved between populations, population-specific modulation of metabolic and signaling pathways may fine-tune larval physiology in response to local environmental pressures. Differences in temperature, salinity, and host availability between the Baltic Sea and Atlantic environments may therefore contribute to divergent transcriptional regulation, supporting ecological adaptation without altering the underlying developmental framework of *A. simplex* s. s. Furthermore, population genetic studies have previously shown that *A. simplex* forms genetically differentiated yet interconnected lineages across European waters, with environmental differences and host structure contributing to observed in this study variability in transcriptomic profiles^4,23,32,63^. Our results provide transcriptomic evidence that such divergence extends beyond neutral genetic variation to include gene expression differences.

The integration of long non-coding RNAs (lncRNAs) into this analysis revealed a rich and previously underappreciated layer of transcriptional regulation in *A. simplex* s. s. Increasing evidence from model systems indicates that lncRNAs modulate gene expression through chromatin regulation, transcriptional interference, and post-transcriptional mechanisms, and the abundance of lncRNA have some level of correlation with parasite development, cellular differentiation, and sex^64,65^. Furthermore, the expression of lncRNA might differ across developmental stages of a parasite or it could be developmentally regulated^66^. For example, sporocysts and adult male and female *Schistosoma mansoni* populations display both shared and stage-specific lncRNA expression profiles during development. Notably, the increased expression of selected lncRNAs in adult worms compared to schistosomula (free-living larval stages) suggests that lncRNAs may contribute to the rapid developmental transition and physiological adaptation of adult *S. mansoni* to parasitism within the mammalian host^65^. Although functional annotations of different classes of RNAs in Anisakis remain limited, emerging studies, such as miRNA from *A. pegreffii*^67^ and lncRNA catalogues from *A. simplex* s. s*.*^45^, suggest that non-coding RNAs may play key roles in parasite development, environmental adaptations and host interactions.

In our dataset, a subset of identified DELs showed detectable associations with DEGs, and the number of DEL–DEG potential interactions varied markedly between comparisons. The Baltic developmental comparison (L4 vs L3) showed the largest and most complex regulatory network, with 630 DELs predicted to regulate 1,696 DEGs, forming 12,312 DEL-DEG potential regulatory relationships, which to our knowledge, is the largest dataset of this type described to date. In contrast, the Atlantic developmental comparison contained a single DEL (MSTRG.540.1), which interacted with 18 different DEGs. These patterns highlight the possible population-specific regulatory deployment of lncRNAs.

The functional annotation of DEGs predicted to be likely regulated by DELs provides insight into the specific biological processes and molecular functions potentially under non-coding RNA control during both developmental and population contrasts. In the population comparison between L4 BAL and L4 ATL larvae, DEL-associated DEGs were significantly enriched in categories related to energy metabolism, ion transport, and structural assemblies. For example, enrichment in calcium ion transport (GO:0006816) is consistent with the established role of calcium signaling in nematode muscle contraction and neuromuscular coordination^68,69^, suggesting that population-dependent modulation of these pathways could influence larval motility or host interaction behaviors. The prominence of oxidoreductase activities (GO:0016491; GO:0016903) among DEL-regulated DEGs aligns with previous reports that redox processes are critical for larval survival and detoxification in response to oxidative stress ^70–72^. In contrast, the developmental comparison between L4 BAL and L3 BAL showed a broader pattern of enriched terms centered on protein biosynthesis, ribonucleoprotein complexes, and metabolic processes, including translation (GO:0006412), ribosomal structure (GO:0005840), and associated enzymatic activities. These findings align with the extensive morphological and physiological changes that occur during larval maturation, where increased translational capacity supports the rapid protein synthesis required for cuticle formation, tissue differentiation, and growth^47^. Enhanced exopeptidase activity (GO:0008238) further implicates post-translational remodeling processes, reflecting observations in other nematode species where proteolytic cascades drive molting and structural reorganization^73,74^. The differential enrichment profiles between population and developmental comparisons suggest that lncRNA-mediated regulation targets distinct biological modules depending on the context. In the population comparison, targeted pathways may reflect local environmental adaptation or population-specific physiological states, whereas in the developmental comparison, lncRNAs appear to interact with core cellular machinery to orchestrate stage transitions. Such context-specific lncRNA roles are increasingly recognized; in *C. elegans* lncRNAs have been shown to be required for normal development and fertility^75^, and stage-specific non-coding RNAs have been implicated in Schistosoma larval transitions^65^.

Collectively, the GO enrichment patterns of DEL-regulated DEGs indicate that long non-coding RNAs are involved in modulating core cellular processes and population-specific physiological pathways in *A. simplex* s. s., extending regulatory complexity beyond protein-coding genes. Such layered transcriptional regulation likely supports developmental progression and contributes to population-level differences associated with contrasting environmental conditions in the Baltic Sea and the Northeast Atlantic. These transcriptional differences may have ecological and biomedical relevance, as several affected pathways are linked to metabolism, structural remodeling, and host interaction, all of which can influence parasite fitness and pathogenic potential in accidental hosts. While regulatory associations provide informative hypotheses, functional validation is required to establish direct regulatory roles of lncRNAs and to disentangle environmental, host-related, and population-specific drivers of expression divergence.

Overall, our transcriptome-wide analysis reveals coordinated developmental and geographic regulation of mRNAs and lncRNAs in *A. simplex* (s. s.), highlighting regulatory mechanisms that may underlie larval adaptation, population differentiation, and host-associated traits relevant to marine ecology and public health.

## Methods

### *Anisakis simplex* larvae

The experimental setup and workflow for comparing the transcriptomes of L3 and L4 developmental stages of *A. simplex* s. s. larvae from two different geographic regions are shown in Fig. 1A.

Parasitic nematodes from the Atlantic region (A) were collected from the biobank established for the PARASITE project (www.parasite-project.eu) at the Institute of Marine Research (IIM-CSIC) in Vigo, Spain. Experiments with *A. simplex* from the Atlantic region were performed using isolated larval stages: L3 (L3 ATL) from Atlantic hake (*Merluccius merluccius*) and L4 (L4 ATL) from striped dolphin (*Stenella coeruleoalba*). All larvae obtained from the biobank of the PARASITE project^76^ were previously taxonomically identified using conventional polymerase chain reaction to amplify the mitochondrial cytochrome c oxidase subunit II gene (mtDNA *COX2*), and the elongation factor *EF1* *α*-1 nuclear DNA gene, as previously described by Levsen et al. (2018)^87^.

Alive L3 stage larvae of *A. simplex* from the Baltic geographical region (BAL) were collected from Baltic herring (*Clupea harengus membras*) caught in the vicinity of Władysławowo (Poland), fishery Władysławowskie, ICES square 383, subdivision 26. All impurities were removed from the 90 individuals. Five of the isolated L3 larvae were subjected to the taxonomic identification by Anis Sensitive Sniper Real-Time PCR kit (A & A Biotechnology, Gdynia, Poland) as described before^77^. To obtain L4 stage larvae the in vitro culture with use of 45 individuals was performed. The larvae were washed several times in a sterile saline solution (0.9% NaCl) and cultured as described previously by Iglesias et al. (2001)^88^. In brief, larvae were washed for 30 min in a bactericidal and fungicidal solution (80 mg of gentamicin sulphate, cat. no. 010807, PPH Galfarm, Kraków, Poland; 0.625 mg of amphotericin B, cat. no. A9528, Sigma Aldrich, Poznań, Poland; 100 mg of chloramphenicol, cat. no. 107464, Pharma Cosmetic, Kraków, Poland; 10.000 IU of penicillin G, cat. no. P3032, Sigma Aldrich, Poznań, Poland, and 4.5 ml of Hanks’ solution, cat. no. H6648, Sigma Aldrich, Poznań, Poland, supplemented to a final volume of 10 ml with 0.9% saline solution). The culture was performed using RPMI-1640 medium (cat. no. R8758, Sigma Aldrich, Poznań, Poland) enriched with 20% fetal bovine serum (cat. no. F7524, Sigma Aldrich, Poznan, Poland) and 1% pepsin (cat. no. P7125, Sigma Aldrich, Poznan, Poland), in a six-well plate (BD Biosciences, Warsaw, Poland), at pH = 4 (maintained with use of 1 M HCl), 5% CO_2_ and 37 °C. The molting to L4 larvae was observed within 6 days in these conditions. The culture medium was renewed every two days, and the larvae were observed every two days for survival assessment under stereomicroscope. All dead larvae were excluded from the further analyses. The L3 stage (L3 BAL) larvae and in vitro-cultured L4 stage larvae (L4 BAL) were store at − 80 °C until further analyses.

### Total RNA isolation, library construction and RNA sequencing

Total RNA was extracted from the larval samples pooled into biological replicates representing four groups (L3 ATL, L3 BAL, L4 ATL, and L4 BAL). Each biological replicate consisted of five larvae pooled prior to RNA extraction. For most groups (L3 ATL, L3 BAL, and L4 BAL), four independent biological replicates were generated (e.g., L3 BAL_1–L3 BAL_4). Due to limited availability of Atlantic L4 larvae from the biobank material, only one biological replicate was available for the L4 ATL group; this replicate also consisted of five pooled larvae.

RNA isolation was performed using TRIzol reagent in combination with the PureLink RNA Mini Kit (cat. no. 12183018A, Invitrogen by Thermo Fisher Scientific, Waltham, Massachusetts, USA), following the manufacturer’s guidelines. RNA concentration and integrity were assessed with the Agilent 2100 Bioanalyzer with the RNA 6000 Nano LabChip Kit (cat. no. 5067-1511, Agilent, CA, USA). Only samples with RNA integrity number (RIN) values above 7.0 were used for library construction, in accordance with the quality criteria applied in previous transcriptomic studies^45^.

For each sample, approximately 5 µg of total RNA was subjected to rRNA depletion using the Ribo-Zero Gold rRNA Removal Kit (cat. no. MRZG12324, Illumina, San Diego, USA). The resulting rRNA-depleted RNA fraction was fragmented using the NEBNext® Magnesium RNA Fragmentation Module (cat. no. E6150S, New England Biolabs, Ipswich, MA, USA) under high-temperature conditions (94 °C for 5–7 min). Fragmented RNA was subsequently reverse transcribed with SuperScript™ II Reverse Transcriptase (cat. no. 1896649, Invitrogen, Waltham, MA, USA), and the generated cDNA was converted into U-labeled second-strand DNA using E. coli DNA polymerase I (cat. no. m0209), RNase H (cat. no. m0297), and dUTP (cat. no. R0133) reagents (New England Biolabs/Thermo Fisher Scientific).

The double-stranded cDNA fragments were end-repaired and A-tailed to facilitate ligation of indexed adapters containing complementary T-overhangs. Adapter-ligated DNA was size-selected to enrich fragments of approximately 300–600 bp using AMPure XP magnetic beads (cat. no. A63881, Beckman Coulter, Indianapolis, Indiana, USA). The U-labeled second strand was selectively degraded with the heat-labile UDG enzyme (cat. no. m0280, New England Biolabs, Ipswich, MA, USA), after which libraries were amplified by PCR under the following conditions: 95 °C for 3 min; 8 cycles of 98 °C for 15 s, 60 °C for 15 s, and 72 °C for 30 s; followed by a final extension at 72 °C for 5 min. The final libraries exhibited an average insert size of 300 ± 50 bp.

Paired-end sequencing (2 × 150 bp, PE150) was performed on the Illumina NovaSeq™ 6000 platform (LC-BioTechnology Co., Ltd., Hangzhou, China) following the manufacturer’s protocol and applying a workflow like that reported previously^45^.

### Bioinformatical analyses

#### RNA-Seq data preprocessing and mapping

The general preprocessing strategy followed standard RNA-Seq workflows used in recent nematode transcriptomic studies, including the approach described by Polak et al. (2025). Raw paired-end reads obtained from Illumina sequencing were processed to remove technical artefacts and low-quality regions prior to downstream analysis. Adapter sequences, polyA/G tails and poor-quality fragments were trimmed using Cutadapt^78^. Reads were discarded if they contained (i) adapter contamination, (ii) polyA or polyG stretches, (iii) more than 5% ambiguous nucleotides (N), or (iv) more than 20% bases with Q ≤ 20. Quality metrics of the filtered datasets, including Q20/Q30 values and GC content, were assessed using FastQC^79^. Reads shorter than 120 nucleotides and with an average Phred quality score below 20 were excluded from the dataset. After filtering, high-quality FASTQ reads were mapped to the *Anisakis simplex* PRJEB496 reference genome from WormBase ParaSite using STAR v.2.7.11.a.^80^. Aligned reads from each sample were assembled into transcript structures using StringTie v2.2.1^81^. To distinguish long non-coding RNAs (lncRNAs) from protein-coding genes, transcripts shorter than 200 bp or those overlapping with known mRNAs were first discarded. Subsequently, the coding potential was evaluated using CPC v.0.9 (score < 0.5) and CNCI v.2.0 (score < 0) algorithms, and only non-coding transcripts with specific class codes (i, j, o, u, x) were identified as lncRNAs.

#### Differentially expressed genes and long non-coding RNAs

Gene and transcript abundances were quantified using StringTie combined with the Ballgown R package^82^. Gene-level FPKM (fragment per kilobase of transcript per million mapped reads) values, where FPKM = [total_exon_fragments/mapped_reads (millions) × exon_length (kB)] for all samples were then quantified using the Ballgown package. Differentially expressed RNAs were identified using the edgeR package (v.4.8.2). Transcripts were considered significantly regulated if they exhibited an absolute log2 fold change ≥ 1 (∣log2 FC∣ ≥ 1) and an adjusted *p* value (p-adj) < 0.05. Based on these criteria, all detected transcripts were grouped into two categories: differentially expressed genes (DEGs) and differentially expressed long non-coding RNAs (DELs). Functional annotations were assigned using the biomaRt package together with ENSEMBL gene information.

#### GO enrichment analysis and pathway enrichment analysis (KEGG)

Gene ontology (GO) and Kyoto Encyclopedia of Genes and Genomes (KEGG)^83^ annotations were obtained utilizing the g:profiler v.0.2.2 R package^84^. Significant genes were assigned to biological processes (BP), cellular components (CC), molecular functions (MF) and KEGG categories. Analysis was performed to discover GO categories or KEGG pathways enriched by DEGs, with an adjusted *p* value threshold of < 0.05.

#### DEL-DEG interactions analysis

Correlations between the expression profiles of DEGs and DELs were assessed using Pearson’s correlation coefficient calculated with the cor function from the stats v. 4.5.1 package in R. Only associations exhibiting an absolute (r) value exceeding 0.99 and an FDR below 0.05 were deemed statistically significant.

### RT-qPCR analysis of selected genes

The mRNA level of selected DEGs was determined by real-time PCR. The primers for chosen genes were designed using the Primer3Plus software^85^ (ELIXIR, Hinxton, Cambridgeshire, UK) and listed in Supplementary Table 1.

The total RNA was isolated previously (see 4.2.) and the cDNA for real-time PCR was obtained with the use of the Applied Biosystems™ High-Capacity cDNA Reverse Transcription Kit (Thermo Fisher Scientific, Vilnius, Lithuania, cat. no. 4374966) according to the manufacturer’s protocol. The real-time PCR was performed with the use of Applied Biosystems™ PowerUp™ SYBR™ Green Master Mix (Thermo Fisher Scientific, Vilnius, Lithuania, cat. no. A25780) according to the manufacturer’s protocol on the QuantStudio™ 3 Real-Time PCR System (Applied Biosystems™, Thermo Fisher Scientific Inc., Waltham, MA, USA). In brief, each reaction contained: 5 μL of master mix (2X), forward and reverse primers in amount of 10 nM of each, 10 ng of cDNA, and a proper volume of nuclease-free water to a final volume of 10 μL. The reactions were performed in four technical replicates for each biological sample. The expression of each gene was calculated using the comparative Pfaffl method^86^, where the expression is presented as the fold change relative to the control, as well as normalized to an endogenous reference gene (*actin*, GenBank Acc. No. KP200883, and *elongation factor 1 alpha 1*, GenBank Acc. No. KP326558) (relative quantification RQ = 1). The results were expressed as Log_2_FC of relative gene expression (means of biological replicates ± SD). Statistical analysis was performed using ANOVA in Prism 8 software (GraphPad Software Inc., San Diego, CA, USA). Significance levels are indicated as * *p* < 0.05, ** *p* < 0.01, and *** *p* < 0.001.

## Supplementary Information

Supplementary Information 1.

Supplementary Information 2.

Supplementary Information 3.

Supplementary Information 4.

Supplementary Information 5.

Supplementary Information 6.

Supplementary Information 7.

Supplementary Information 8.

Supplementary Information 9.

Supplementary Information 10.

## Data Availability

The sequencing data generated for this study can be found in the European Nucleotide Archive (ENA); accession no. PRJEB100984.
