# Correction: Developmental and geographic transcriptomic variation in *Anisakis simplex* (s. s.) reveals lncRNA-mediated regulation of mRNA expression

**DOI:** 10.1038/s41598-026-62600-5

**Published:** 2026-07-21

**Authors:** Robert Stryiński, Mateusz Maździarz, Mónica Carrera, Elżbieta Łopieńska-Biernat

**Affiliations:** 1 Department of Biochemistry, Faculty of Biology and Biotechnology, University of Warmia and Mazury in Olsztyn, Olsztyn, Poland; 2 Department of Botany and Nature Protection, Faculty of Biology and Biotechnology, University of Warmia and Mazury in Olsztyn, Olsztyn, Poland; 3 Department of Food Technology, Institute of Marine Research (IIM), Spanish National Research Council (CSIC), Vigo, Spain

Correction to: *Scientific Reports* 10.1038/s41598-026-47984-8, published online 20 April 2026

The original version of this Article contained an error in panel E of Figure 2, where three partial bars incorrectly appear above the ‘1’ and ‘0’ for ‘ATL L4 vs ATL L3’ in the x-axis of the graph. Furthermore, a partial bar incorrectly appears within the ‘304’ bar for ‘BAL L4 vs BAL L3’. The original Fig. 2 and accompanying legend appear below.

**Fig. 2:**
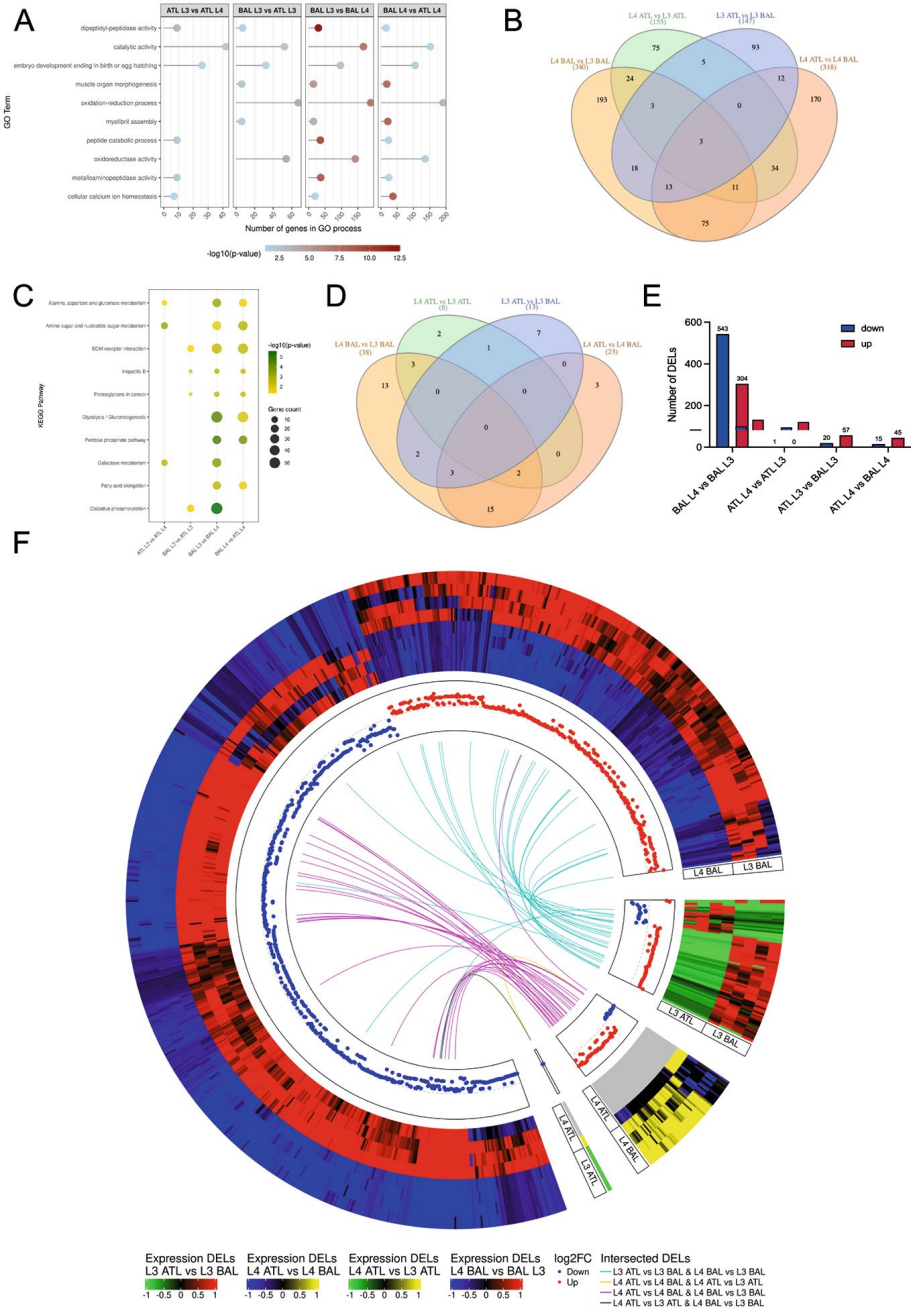
Functional enrichment of DEGs and expression patterns of DELs in *A. simplex* s. s. larvae. (**A**) Dot plot summarizing Gene Ontology (GO) enrichment analysis of DEGs identified in four pairwise comparisons: L4 BAL vs L3 BAL, L4 ATL vs L3 ATL, L3 ATL vs L3 BAL, and L4 ATL vs L4 BAL. The x-axis represents the number of DEGs assigned to each GO term, while color intensity indicates statistical significance expressed as –log_10_ (p value). Enrichment includes terms from the biological process (BP), molecular function (MF), and cellular component (CC) categories. Details can be found in Supplementary File 2. (**B**) Venn diagram illustrating the overlap of significantly enriched GO terms among the four DEG comparisons. Only three GO terms: catalytic activity (GO:0003824), dipeptidyl-peptidase activity (GO:0008239), and embryo development ending in birth or egg hatching (GO:0009792), were shared across all comparisons, whereas the majority of significant GO terms were comparison-specific. (**C**) KEGG pathway enrichment analysis of DEGs across the four pairwise comparisons. Dot size corresponds to the number of DEGs mapped to each pathway, and color intensity represents pathway significance (–log_10_ (p value)). Selected enriched pathways are shown for each comparison. Details can be found in Supplementary File 3. (**D**) Venn diagram showing the distribution of significantly enriched KEGG pathways across comparisons. No KEGG pathway was shared among all four datasets, indicating distinct pathway-level responses associated with larval developmental stage and geographical origin. (**E**) Numbers of significantly upregulated and downregulated DELs identified in each comparison. Differential expression was defined as |log_2_ FC|≥ 1 with an adjusted *p* value < 0.05. (**F**) Circos plot integrating expression profiles and shared DELs across all comparisons. Outer heatmap tracks represent normalized DEL expression levels, with red and blue indicating upregulation and downregulation, respectively. Inner tracks show log_2_ FC values, and connecting links indicate DELs shared between comparisons related to larval stage and geographical population.

The original Article has been corrected.

