# Supplementary figures and images for: Developmental and geographic transcriptomic variation in Anisakis simplex (s. s.) reveals lncRNA-mediated regulation of mRNA expression
Source: Sci Rep. 2026 Apr 20;16:18383. doi: 10.1038/s41598-026-47984-8 (PMC13266063; doi:10.1038/s41598-026-47984-8)

Relative gene expression (Pfaffl method, mean  $\pm$  SD)

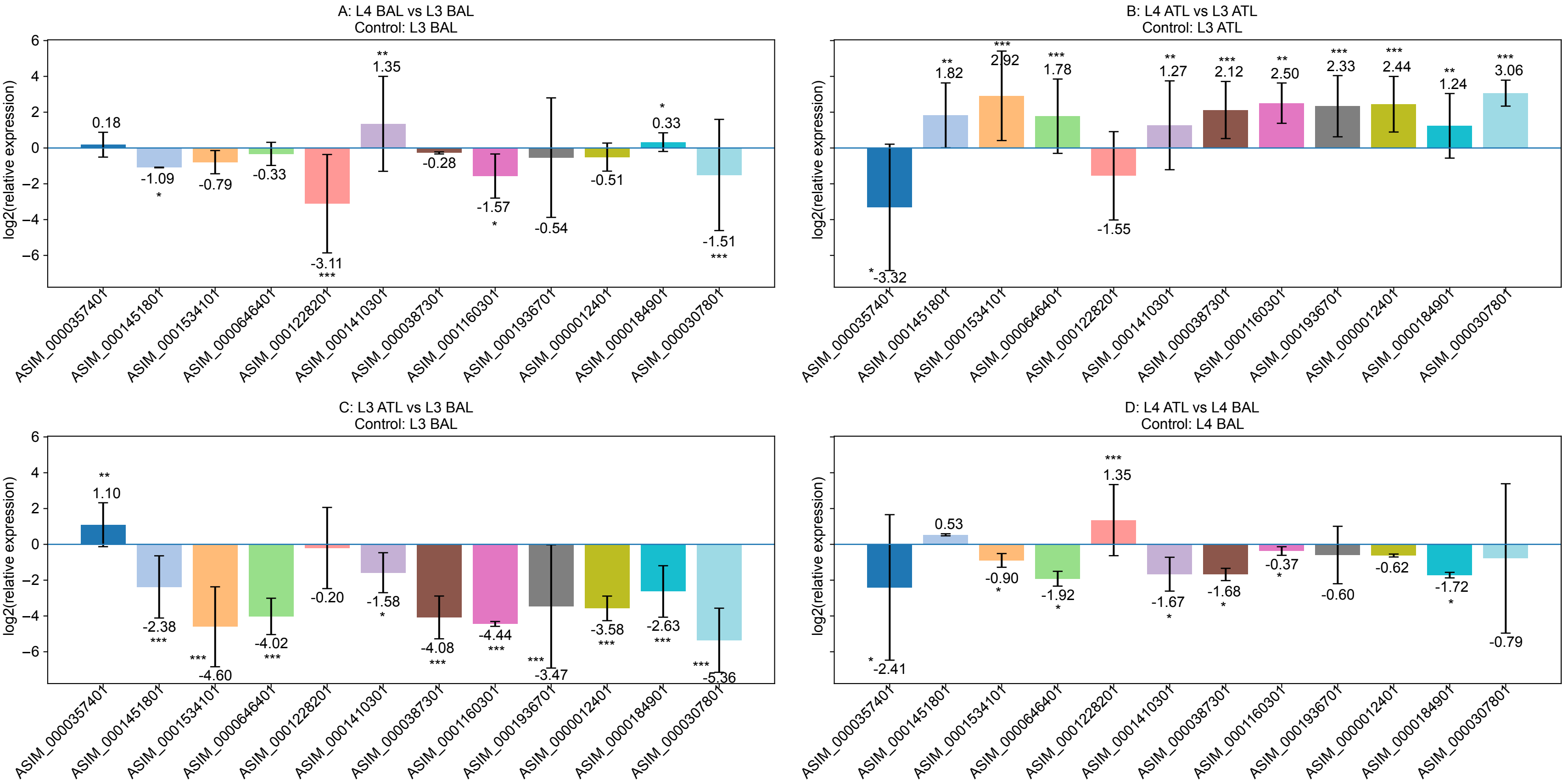

Supplement: Supplementary file 1 — Supplementary Information 1. [file 41598_2026_47984_MOESM1_ESM.pdf]
