# Supplementary material for: Developmental and geographic transcriptomic variation in Anisakis simplex (s. s.) reveals lncRNA-mediated regulation of mRNA expression
Source: Sci Rep. 2026 Apr 20;16:18383. doi: 10.1038/s41598-026-47984-8 (PMC13266063; doi:10.1038/s41598-026-47984-8)
Supplement: Supplementary file 2 — Supplementary Information 2. [file 41598_2026_47984_MOESM2_ESM.pdf]

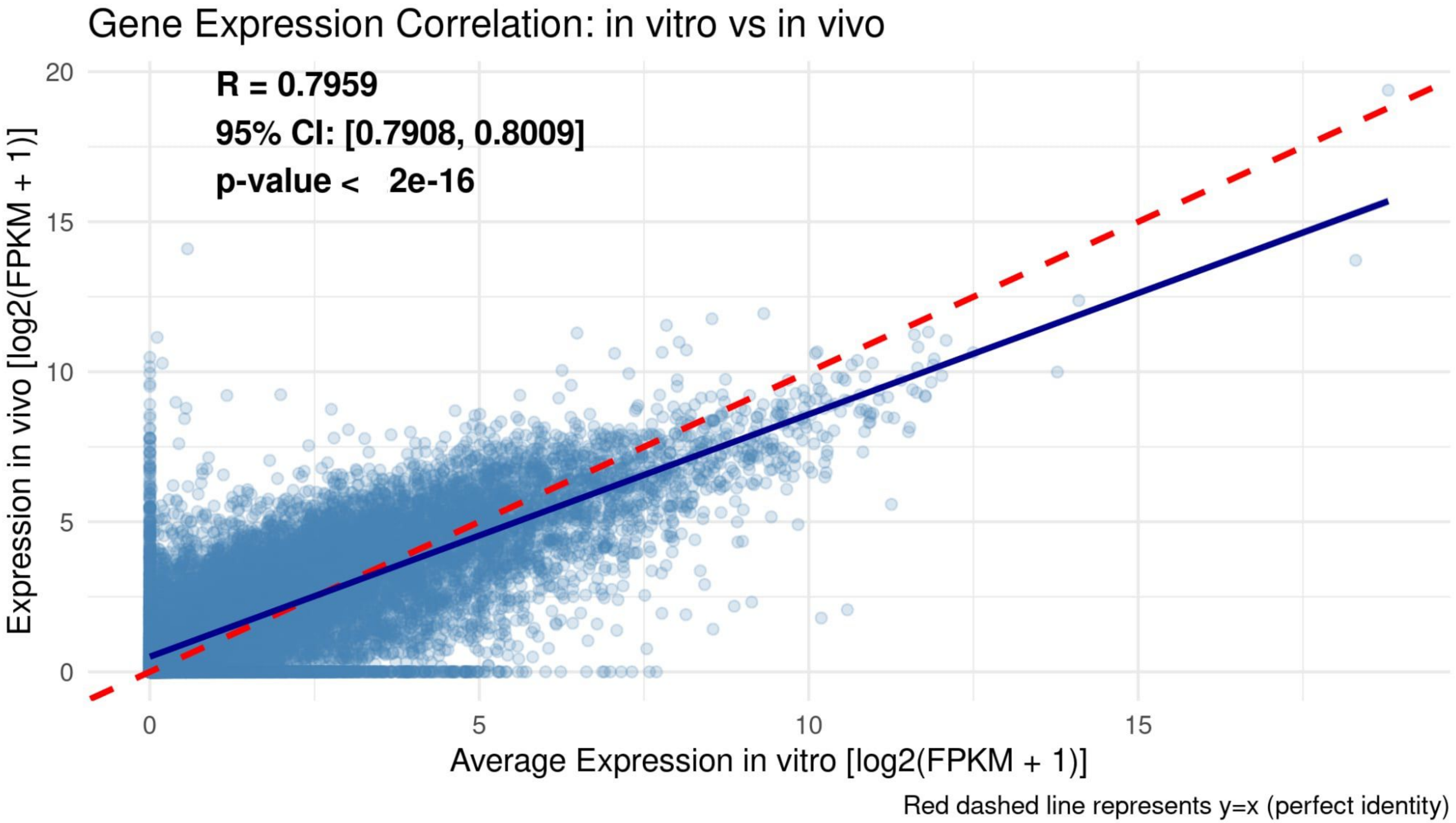

Gene Expression Correlation: in vitro vs in vivo
R = 0.7959
95% CI: [0.7908, 0.8009]
p-value < 2e-16
Expression in vivo [log2(FPKM + 1)]
Average Expression in vitro [log2(FPKM + 1)]
0
5
10
15
20
Red dashed line represents y=x (perfect identity)
