# Supplementary material for: Developmental and geographic transcriptomic variation in Anisakis simplex (s. s.) reveals lncRNA-mediated regulation of mRNA expression
Source: Sci Rep. 2026 Apr 20;16:18383. doi: 10.1038/s41598-026-47984-8 (PMC13266063; doi:10.1038/s41598-026-47984-8)
Supplement: Supplementary file 9 — Supplementary Information 9. [file 41598_2026_47984_MOESM9_ESM.docx]

Table S1. The list of primers used for Real-time PCR.

| Name | No. accession GenBank | Forward sequence:(5'to3') | Reverse sequence:(5'to3') |
| --- | --- | --- | --- |
| *NA* | ASIM_0000357401 | GTCGCTCTTCATCCGTTTCC | CGTGAGTGACCGGTGAGTAT |
| *Vinculin* | ASIM_0001451801 | CGTGAATTCTCGGATGCGTT | TCCGAGTTGTCAGCCTCATT |
| *Sugar transporter SWEET* | ASIM_0001534101 | TTGATACGATGAGTGCCGGT | TGCAGAATCGACGAAAGCTG |
| *Alpha-1,4 glucan phosphorylase* | ASIM_0000646401 | CGTTGGCCAGTTTCGTTGAT | TTCGAGTGTAATGCAGCCAC |
| *26S proteasome regulatory subunit 7* | ASIM_0001228201 | TGCAGCGAACAATGTTGGAA | CGCTCTGCCTGCTAAATCAG |
| *Ani s 4 allergen* | ASIM_0001410301 | TAGCGGTGGTTGTCTTTTGC | TTGCAGACACCACTTTGACG |
| *Secreted protein* | ASIM_0000387301 | ATGAAGCTCTGCATCTTGGC | CTGCTTCGGTTTTGTCTGGG |
| *Troponin-like protein* | ASIM_0001160301 | TCGATGAGTTTTGCGCACTT | ATTTCATCGACGGCTGCTTC |
| *PGM_PMM_I domain-containing protein* | ASIM_0001936701 | GGTCGTCATAACAGTCGCAG | TTGGATTGTGTGATGCCGTG |
| *FAM184 domain-containing protein* | ASIM_0000012401 | ATGAATTGCCTGCGGTTGTT | ATCACAGATTGCCGTCGTTG |
| *Probable 26S protease regulatory subunit 6B* | ASIM_0000184901 | ATCGTGAGCTGTTGAAACCG | TCACGTACCTCCTGCTTCTG |
| *Actin* | KP200883 | TGGAGTGGTGCTTGACTCAG | TCACGAACAATCTCACGCTC |
| *Elongation factor1alpha1* | KP326558 | TCCTCAAGCGTTGTTATCTGTT | AGTTTTGCCACTAGCGGTTCC |
