# Supplementary material for: Developmental and geographic transcriptomic variation in Anisakis simplex (s. s.) reveals lncRNA-mediated regulation of mRNA expression
Source: Sci Rep. 2026 Apr 20;16:18383. doi: 10.1038/s41598-026-47984-8 (PMC13266063; doi:10.1038/s41598-026-47984-8)
Supplement: Supplementary file 10 — Supplementary Information 10. [file 41598_2026_47984_MOESM10_ESM.docx]

Supplementary Files:

**Supplementary Table S1.** The list of primers used for Real-time PCR.

**Supplementary File 1.** List of differentially expressed genes (DEGs) identified in all four pairwise comparisons, including gene identifiers, log₂ fold change values, and FDR-adjusted p values.

**Supplementary File 2.** Gene Ontology (GO) enrichment analysis results for differentially expressed genes (DEGs) across all pairwise comparisons, including GO term IDs, functional categories (BP, MF, CC), gene counts, and statistical significance.

**Supplementary File 3.** KEGG pathway enrichment analysis of differentially expressed genes (DEGs) for all pairwise comparisons, including pathway identifiers, gene counts, and significance values.

**Supplementary File 4.** List of differentially expressed long non-coding RNAs (DELs) identified in all pairwise comparisons, including expression changes and statistical significance.

**Supplementary File 5.** Correlation analysis results for predicted DEL–DEG regulatory interactions across all comparisons, including correlation coefficients and associated target genes.

**Supplementary File 6.** Gene Ontology (GO) enrichment analysis results for DEGs that were predicted to be regulated by DELs, including GO term IDs, functional categories (BP, MF, CC), gene counts, and statistical significance.

Supplementary Figures:

**Supplementary Figure 1. Differential expression of selected genes across developmental stages and populations.** Relative expression of selected genes was calculated using the Pfaffl method and is shown as log₂ fold change relative to the appropriate control. Panels illustrate comparisons between developmental stages within the Baltic population (A, L4 BAL vs L3 BAL), developmental stages within the Atlantic population (B, L4 ATL vs L3 ATL), population-specific differences at the L3 stage (C, L3 ATL vs L3 BAL), and population-specific differences at the L4 stage (D, L4 ATL vs L4 BAL). *Actin* and *EF-1 alpha* were used as the reference genes. Bars represent mean log₂-transformed Pfaffl values calculated from biological replicates (n = 4), and error bars indicate the standard deviation calculated from these log₂-transformed Pfaffl values. Each gene is depicted using a consistent color across all panels, and numeric values above bars indicate mean log₂ fold change. Statistical significance was assessed using a one-sample t-test on log₂-transformed Pfaffl values relative to the corresponding control, followed by Benjamini–Hochberg false discovery rate (FDR) correction applied separately for each panel. Significance levels are indicated as * *p* < 0.05, ** *p* < 0.01, and *** *p* < 0.001.

**Supplementary Figure 2. Correlation of gene expression profiles between in vitro–derived and in vivo L4 larvae of *Anisakis simplex* s. s.** Scatter plot showing the relationship between gene expression levels in L4 larvae obtained from an in vitro culture system and L4 larvae collected from a natural host (Atlantic population, striped dolphin-derived biobank material). Each point represents an individual transcript. A strong positive correlation was observed between the two datasets (Pearson correlation coefficient R = 0.7959; p < 2 × 10⁻¹⁶), indicating a high degree of similarity in global expression patterns. These results support the biological relevance of the in vitro model and demonstrate that it captures a substantial portion of the transcriptional landscape observed in naturally developed L4 larvae.
